# 2-(4-Fluorophenyl)-N-phenylacetamide Derivatives as Anticancer Agents: Synthesis and *In-vitro* Cytotoxicity Evaluation 

**Published:** 2013

**Authors:** Alireza Aliabadi, Sajad Andisheh, Zahra Tayarani-Najaran, Mona Tayarani-Najaran

**Affiliations:** a *Department of Medicinal Chemistry, Faculty of Pharmacy, Kermanshah University of Medical Sciences, Kermanshah, Iran.*; b *Students Research Committee, Kermanshah University of Medical Sciences, Kermanshah, Iran.*; c *Department of Pharmacodynamics and Toxicology, School of Pharmacy, Mashhad University of Medical Sciences, Mashhad, Iran.*; d *Departmrnt of Chemistry, Alzahra University, Tehran, Iran. *

**Keywords:** Synthesis, Phenylacetamide derivatives, Anticancer, Cytotoxicity, MTS assay

## Abstract

Cancer is a major global problem and is the second leading cause of mortality in the developed countries.Resistance to current chemotherapeutics and high incidence of adverse effects are the two principal reasons for developing new anticancer agents. Phenylacetamide derivatives can act as potential anticancer agents. Synthesis and screening of 2-(4-Fluorophenyl)- *N*-phenylacetamide derivatives in present study showed that these compounds act as potent anticancer agents especially against PC3(prostate carcinoma) cell line. Compounds 2a-2c with nitro moiety demonstrated a higher cytotoxic effect than compounds 2d-2f with methoxy moiety. All compounds in this series exhibited lower activity than imatinib as reference drug. Compounds 2b (IC_50_ = 52 μM) and 2c (IC_50 _= 80 μM) were the most active compounds against PC3 cell line in comparison with imatinib(IC_50 _= 40 μM). Compound 2c (IC_50_ = 100 μM) with *p*-nitro substituent was the most active compound compared to imatinib(IC_50_ = 98 μM) in MCF-7 cell line.

## Introduction

Cancer is a major global problem and is the second leading cause of mortality in the developed countries. Since many of the current pharmacotherapeutic methods have problems with toxicity and drug-resistance, there is a strong demand for the discovery and development of effective, saferand novelcancer therapies (1). 

Since it is known that the anti-tumor efficacy of many chemotherapeutic agents are correlated with their ability to induce apoptosis, novel approaches to promote apoptosis in cancer cells via targeting regulators of apoptosis could lead to the development of new anticancer treatments. In addition, these new agents may overcome tumor resistance to the conventional anti-cancer drugs (2-9). 

**Figure. 1:**
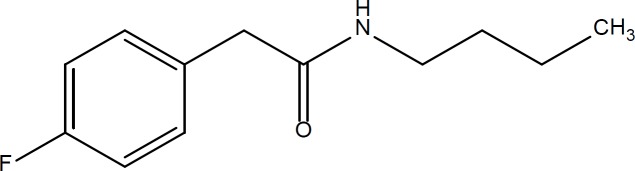
Structure of 4-Fluoro-*N*-butylphenylacetamide as potent anticancer lead compound

Phenylacetate (PA) and related aromatic fatty acids have been shown to possess anti-proliferative and differentiating effects on various human cancer cell lines such as glioblastomas, leukemias, prostate carcinomas, and breast carcinomas. A phase I clinical trial with PA has also proved *in-vivo *anti-tumor activity in humans. Moreover, it has been reported that PA induces the differentiation process together with apoptosis *in-vitro *and *in-vivo*. The ability of PA to induce tumor growth inhibition, differentiation, and apoptosis of cancer cells, along with the relatively low toxicity at clinically effective doses, prompted us to find a more potent analogue of PA. Recently, several derivatives of phenyl acetic acid were synthesized and reported as potential anticancer agents (Figure 1) (10, 11). 

**Figure 2:**
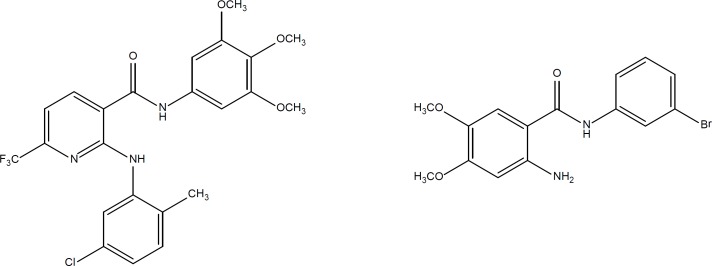
Structure of two aniline derivatives as potent anticancer lead compounds

**Figure 3:**
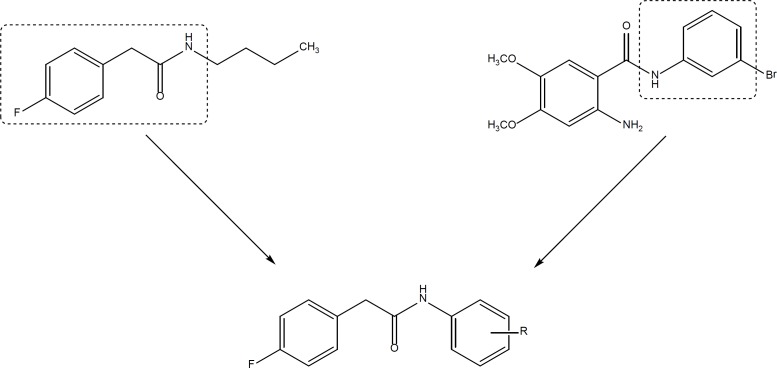
Design of 2-(4-Fluorophenyl)-*N*-phenylacetamide derivatives

On the other hand, aniline derivatives are also potent *in-vitro *inhibitors of cell proliferation and growth (Figure 2) (12-14). In the present project, we combined the structure of phenylacetamide and anilide derivatives for designing of new anticancer agents (Figure 3). 

Compounds 2a-2f were tested against three cancerous cell lines using MTS assay. Cell lines used in this study werePC3(prostate carcinoma), MCF-7(breast cancer) and HL- 60(promyelocytic leukemia) (Table 1). Totally, all tested compounds showed a better cytotoxic activity toward the PC3 cell line in comparison with other cell lines. On the other hand, MCF-7 cell line was the most resistant cell line to the tested compounds. Compounds 2a- 2c with nitro moiety demonstrated a higher cytotoxic effect than compounds 2d-2f with methoxy moiety. All compounds in this series exhibited lower activity than imatinib as reference drug. Compounds 2b (IC_50_ = 52 μM) and 2c (IC_50 _= 80 μM) were the most active compounds against PC3 cell line in comparison with imatinib (IC_50_ = 40 μM). Compound 2c (IC_50_ = 100 μM) with *p*-nitro substituent was the most active compound compared to imatinib(IC_50_ = 98 μM) in MCF-7 cell line. 

**Table 1:** Cytotoxicity results(IC_50_, μM) of compounds 2a-2g in comparison with imatinib

	**2a**	**2b**	**2c**	**2d**	**2e**	**2f**	**2g**	**Imatinib**
	*o*-NO_2_	*m*-NO_2_	*p*-NO_2_	*o*-OCH_3_	*m*-OCH_3_	*p*-OCH_3_	H	
PC3	196	**52**	**80**	158	156	168	250<	40
MCF-7	250<	191	250<	250<	247	250<	250<	79
HL-60	208	178	**100**	218	206	243	250<	98

## Experimental

*General procedure for the synthesis of compounds 2a-2g*

According to the Figure 3, for the preparation of compounds 2a-2g, equimolar quantities of 4-fluorophenyl acetic acid, EDC and HOBt were mixed and stirred in acetonitrile for 30 min. Then, the appropriate aniline derivatives were added and stirring was continued for 24 h. The completion of the reaction was checked by thin layer chromatography. The acetonitrile was evaporated and water/ethyl acetate was added. Ethyl acetate phase was separated and washed two times by sodium bicarbonate, diluted sulfuric acid and brine. The organic layer was dried by anhydrous sodium sulfate and filtered. The ethyl acetate was evaporated under reduced pressure using rotary evaporator (14). 

*2-(4-Fluorophenyl)-N-(2-nitrophenyl) acetamide (2a)*

mp: 122-129 °C, Yield: 65%, ^1^H NMR (CDCl_3_, 400 MHz) *δ*: 3.63 (s, 2H, -CH_2_-), 6.69(t, 1H, *J *= 8Hz, H_4_-2-Nitrophenyl), 6.8 (d, 1H, *J *= 8Hz, H_6_-2-Nitrophenyl), 7.2 (t, 1H, *J *= 8Hz, H_3_-2-Nitrophenyl), 7.54 (t, 2H, *J *= 8Hz, H_2,6_, 4-Fluorophenyl), 7.89(t, 2H, *J *= 8Hz, H_3,5_, 4-Fluorophenyl), 8.11(d, 1H, *J *= 8Hz, H_3_-2- Nitrophenyl), 10.15 (brs, 1H, NH). IR(KBr, cm^- 1^) ῡ: 3475, 3344, 1624, 1494, 1431, 1342, 1220, 1157, 720. MS(m/z, %): 274(M^+^, 25), 136(75), 109(100), 83(20), 63(10). 

*2-(4-Fluorophenyl)-N-(3-nitrophenyl) acetamide(2b)*

mp: 138 °C, Yield: 57%,^1^H NMR (CDCl_3_, 400 MHz) *δ*: 3.76 (s, 2H, -CH_2_-), 6.65 (d, 2H, *J *= 8Hz, 4-Fluorophenyl), 6.85 (d, 2H, *J *= 8Hz, 4-Fluorophenyl), 7.07 (t, 1H, *J *= 8Hz, H_5_-3-Nitrorophenyl), 7.15 (d, 1H, H_6_-3- Nitrophenyl), 7.26 (d, 1H, H_4_-3-Nitrophenyl), 7.29(s, 1H, H_2_-3-Nitrophenyl), 10.15 (brs, 1H, NH). IR(KBr, cm^-1^) δ: 2922, 1624, 1523, 1508, 1344, 1259, 731. MS(m/z, %): 274(M^+^, 20), 136(85), 109(100), 83(22), 63(8). 

*2-(4-Fluorophenyl)-N-(4-nitrophenyl) acetamide(2c)*

mp: 123 °C, Yield: 72%,^1^H NMR (CDCl_3_, 400 MHz) *δ*:3.76(s, 2H, -CH_2_CO-), 6.62(d, 2H, *J *= 8Hz, 4-Flurophenyl), 7.62(d, 2H, *J *= 8Hz, 4-Fluorophenyl), 8.07(d, 2H, *J *= 8Hz, H_2,6_ , 4-Nitrophenyl), 8.17(d, 2H, *J *= 8 Hz, H_3,5_ , 4-Nitrophenyl), 10.16 (brs, 1H, NH). IR(KBr, cm^-1^) *δ: *3340, 1712, 1597, 1543, 1500, 1338, 1253, 1147, 1111, 858, 750. MS(m/z, %): 274(M^+^, 20), 136(80), 109(100), 83(25), 63(10). 

*2-(4-Fluorophenyl)-N-(2-methoxyphenyl) acetamide(2d)*

mp: 98 °C, Yield: 61%,^1^H NMR (CDCl_3_, 400 MHz) *δ*: 3.72 (s, 2H, -CH_2_-), 3.76 (s, 3H, -OCH_3_), 6.81 (d, 1H, *J *= 8 Hz, H_3_-2-Methoxyphenyl), 6.94 (t, 1H, *J *= 8Hz, H_4_-2-Methoxyphenyl), 7.01 (t, 1H, *J *= 8Hz, H_5_-2-Methoxyphenyl), 7.06(t, 2H, *J *= 8Hz, H_2,6_-4-Fluorophenyl), 7.30(t, 2H, *J *= 8Hz, H_3,5_-4-Fluorophenyl), 7.76 (brs, 1H, NH), 8.33 (d, 1H, *J *= 8 Hz, H_6_-2-Methoxyphenyl). IR (KBr, cm^-1^) *δ*: 3282, 2900, 2860, 1662, 1596, 1537, 1510, 1500, 1460, 1436, 1409, 1342, 1259, 1232, 1215, 1182, 1157, 1114, 1031, 950, 752. MS(m/z, %): 259(M^+^, 35), 123(65), 109(100), 83(30). 

*2-(4-Fluorophenyl)-N-(3-methoxyphenyl) acetamide(2e)*

mp:108-110 °C, Yield: 56 %,^1^H NMR (CDCl_3_, 400 MHz) *δ*: 3.69 (s, 2H, -CH_2_-), 3.77 (s, 3H, -OCH_3_), 6.64 (d, 1H, *J *= 8 Hz, H_4_-3-Methoxyphenyl), 6.84 (d, 1H, *J *= 8 Hz, H_6_-3-Methoxyphenyl), 7.08(m, 3H, aromatic), 7.16(t, 2H, *J *= 8Hz, 4-Fluorophenyl), 7.25(t, 2H, *J *= 8Hz, 4-Fluorophenyl), 7.6 (brs, 1H, NH).IR(KBr, cm^-1^) *δ*: 3260, 1664, 1597, 1533, 1510, 1452, 1429, 1288, 1219, 1155, 1035, 779. MS(m/z, %): 259(M^+^, 20), 150(90), 123(85), 109(100), 92(40), 83(30), 77(40), 52(30). 

*2-(4-Fluorophenyl)-N-(4-methoxyphenyl)acetamide(2f)*

mp:142-148 °C, Yield: 78%,^1^H NMR (DMSO-d_6_, 400 MHz) *δ*: 3.68 (s, 2H, -CH_2_), 3.77 (s, 3H, -OCH_3_), 6.82 (d, 2H, *J *= 8 Hz, H_3,5_, 4-Methoxyphenyl), 7.06 (t, 2H, H_3,5_, 4-Fluorophenyl), 7.30 (t, 2H, H_2,6_, 4-Fluorophenyl), 7.36 (d, 2H, *J *= 8 Hz, H_2,6_, 4-Methoxyphenyl), 7.55 (brs, 1H, NH). IR(KBr, cm^-1^) *δ*: 3300, 1651, 1604, 1539, 1506, 1492, 1452, 1436, 1409, 1246, 1234, 1219, 1157, 1031, 827, 754. MS(m/z, %): 259(M^+^, 40), 123(100), 108(95), 83(20).

*2-(4-Fluorophenyl)-N-phenylacetamide(2g)*

mp:131-134 °C, Yield: 69%,^1^H NMR (DMSO-d_6_, 400 MHz) *δ*: 3.63(s, 2H, -CH2-), 7.03(t, 1H, *J *= 8 Hz, H_4_-phenyl), 7.15(t, 2H, *J *= 8 Hz, H_2,6_-4-Fluorophenyl), 7.29(t, 2H, *J *= 8 Hz, H_3,5_, 4-Fluorophenyl), 7.36(t, 2H, *J *= 8 Hz, H_3,5_-Phenyl), 7.58(d, 2H, *J *= 8 Hz, H_2,6_-Phenyl). IR(KBr, cm^-1^) *δ*: 3284, 1654, 1598, 1546, 1508, 1487, 1442, 1220, 1130, 752. MS(m/z, %): 229(M^+^, 20), 136(10), 120(40), 109(95), 93(100), 83(40), 77(20), 65(30).

**Figure 4:**
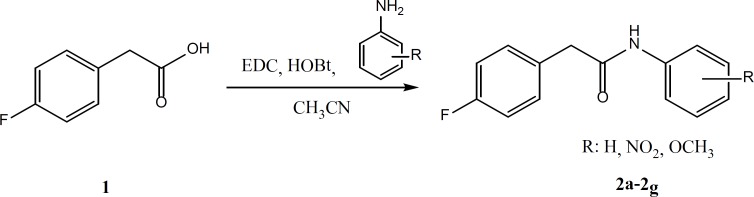
Synthesis of compounds2a-2g

*Cytotoxicity assay*

The MTS assay is based on the reduction by mitochondrial dehydrogenase in metabolically active cells of the novel tetrazolium compound, MTS, to the water-soluble formazan that absorbs at 490 nm. PC3, MCF7 and HL-60 cells were seeded in each well of a 96-microwell plate and treated with various concentrations of the compound(s) to be tested. After incubation for 48 h, Cell Titer 96 Aqueous One Solution Reagent (Promega, Madison, WI), which is composed of the novel tetrazolium compound MTS and an electron coupling reagent phenazineethosulfate (PES, a redox intermediary), was added to each well according to the manufacturer’s instructions. After 3 h, cell viability was determined by measuring the absorbance at the wavelength of 490 nm using an ELISA microplate reader (Awareness, Palm City, FL). The cytotoxicity of the compound(s) was presented as mean of 3 independent experiments with 3 replicates for each compound (s) concentration (15).
